# Supplementary material for: Fingerprinting, Antimicrobial, Antioxidant, Anticancer, Cyclooxygenase and Metabolic Enzymes Inhibitory Characteristic Evaluations of Stachys viticina Boiss. Essential Oil
Source: Molecules. 2019 Oct 28;24(21):3880. doi: 10.3390/molecules24213880 (PMC6864729; doi:10.3390/molecules24213880)
Supplement: Supplementary file 1 [file molecules-24-03880-s001.pdf]

Supplementary material Figures S1-S5

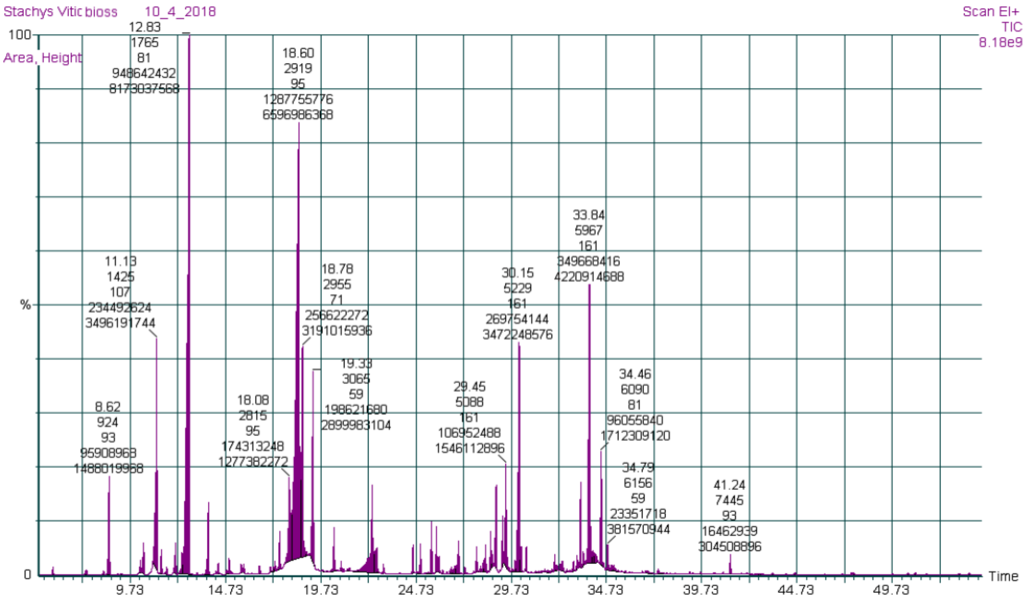

Figure 1. GC-MS chromatogram of S. viticina EO.

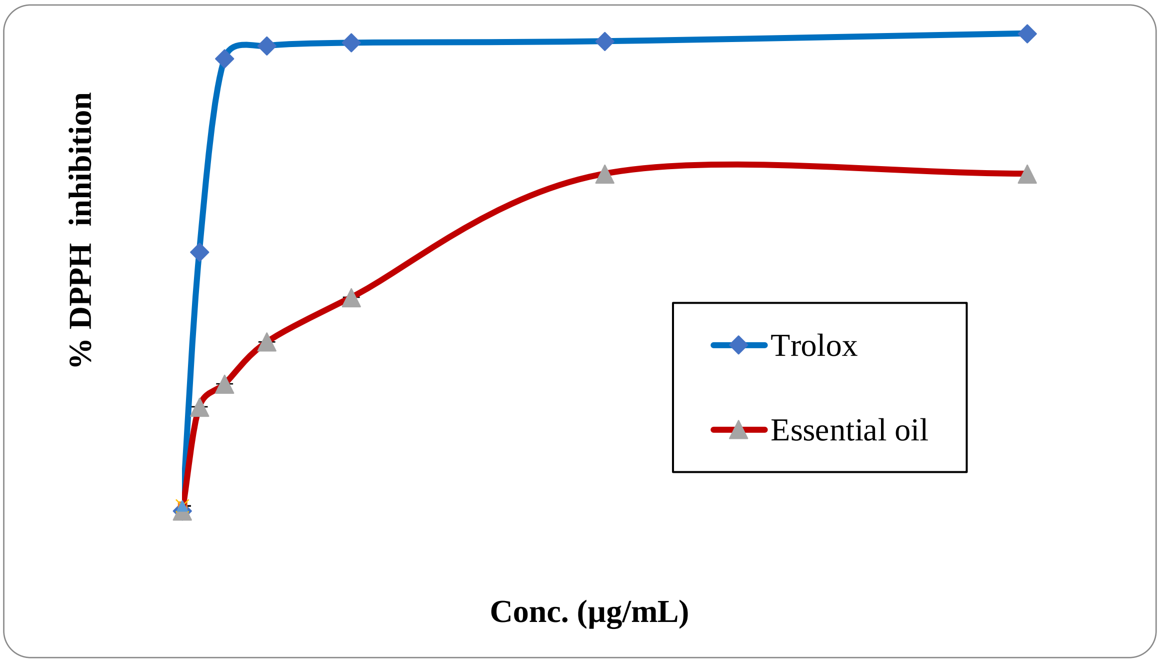

Figure 2. DPPH Inhibition % by Trolox (positive control) and S. viticina EO.

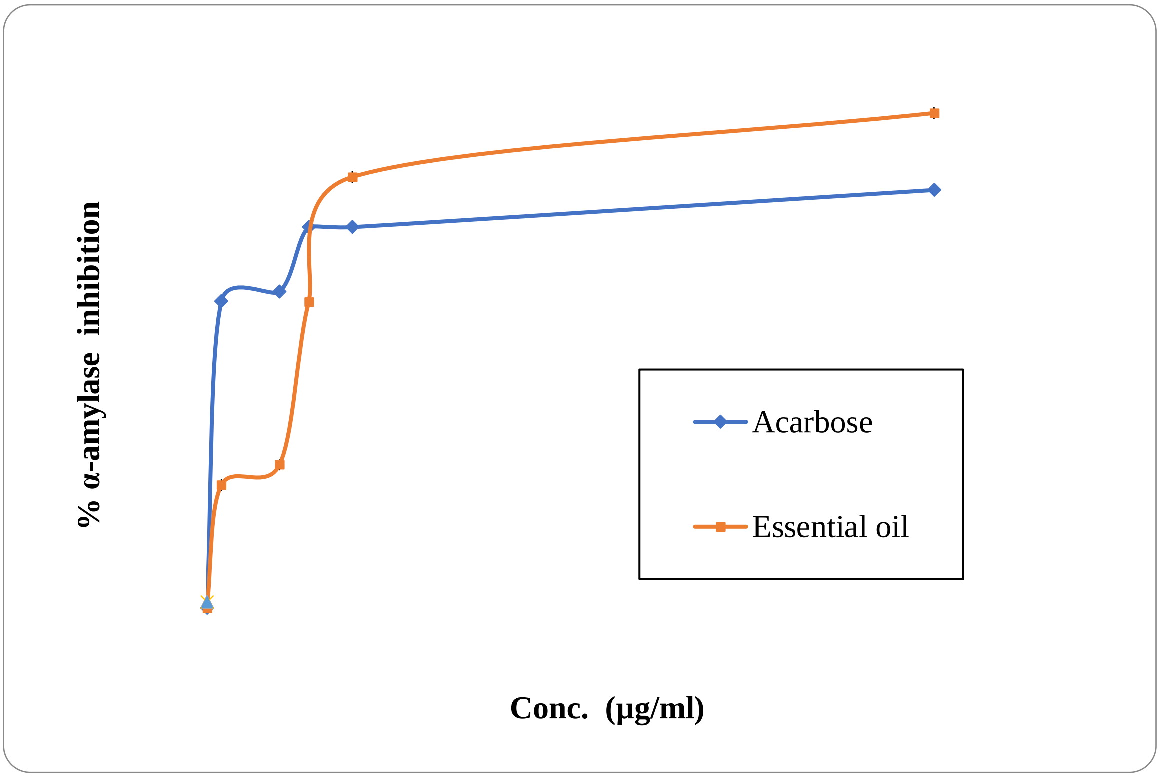

Figure 3. The α-amylase inhibitory potential by Acarbose (positive control) and S. viticina EO.

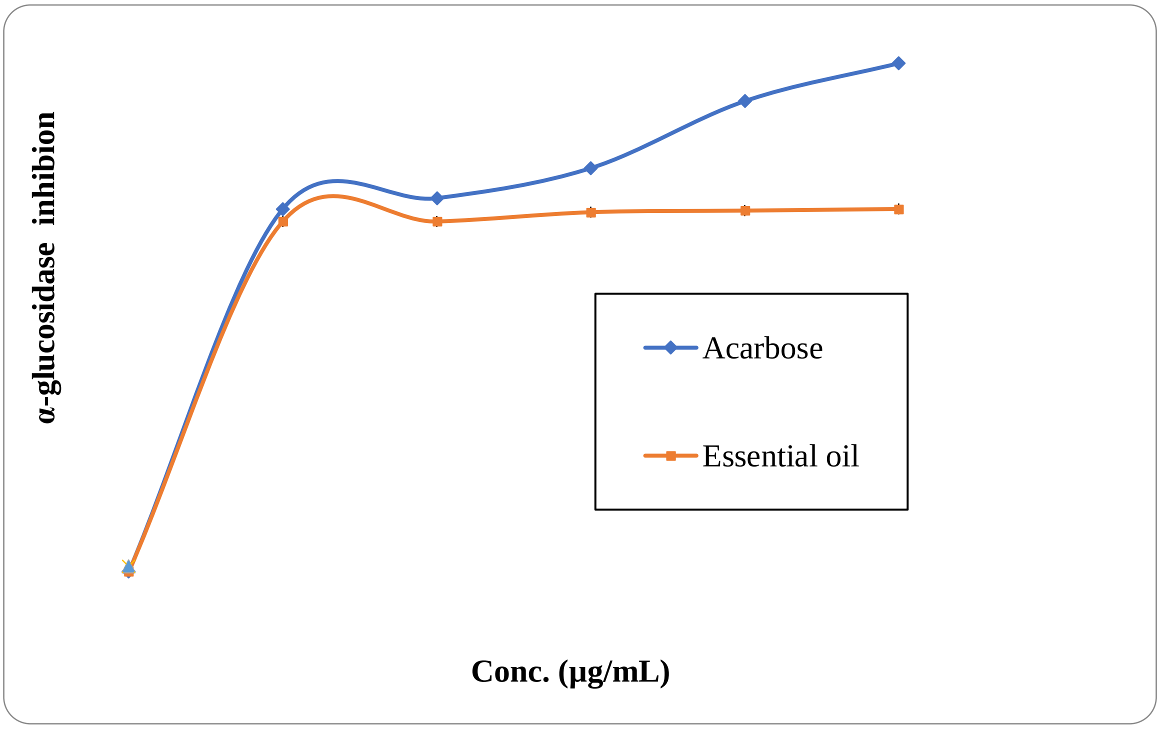

Figure 4. The α-glucosidase inhibitory potential by Acarbose (positive control) and S. viticina EO.

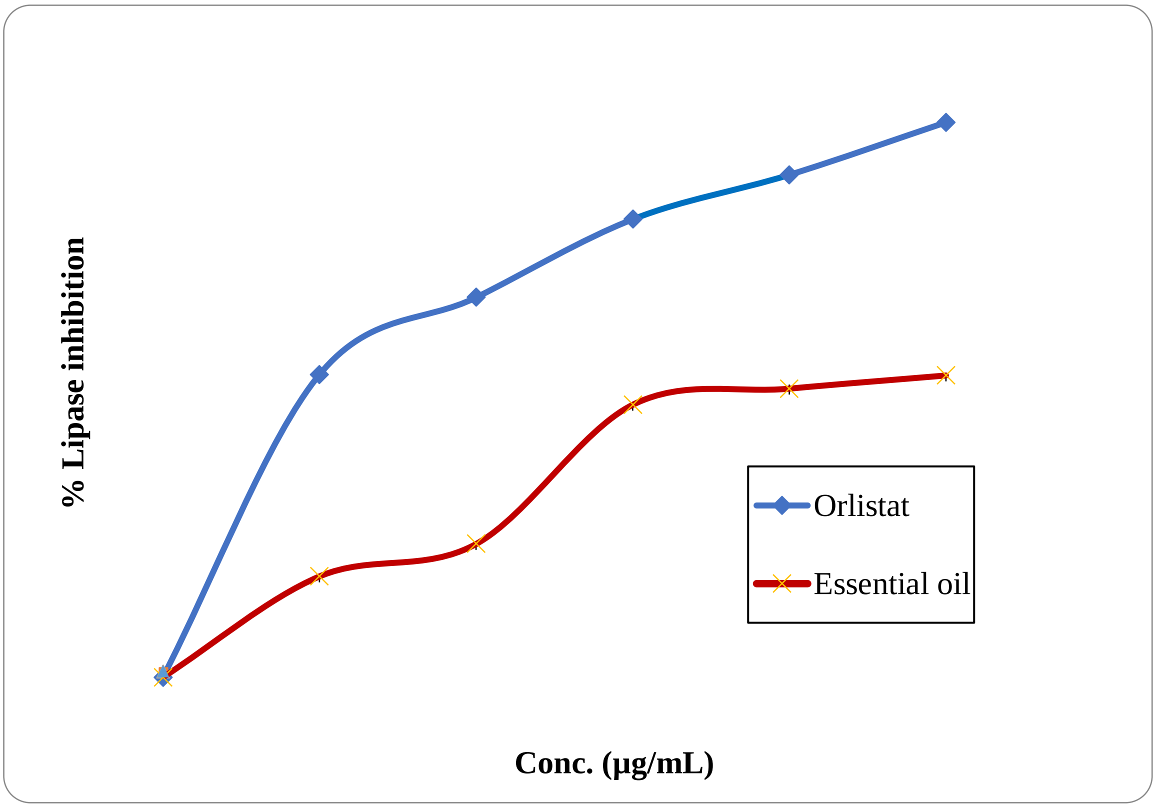

Figure 5. Lipase enzyme inhibitory activity by S. viticina EO and Orlistat (positive control).
